# Identification of Human Plasma Metabolites Exhibiting Time-of-Day Variation Using an Untargeted Liquid Chromatography–Mass Spectrometry Metabolomic Approach

**DOI:** 10.3109/07420528.2012.699122

**Published:** 2012-07-23

**Authors:** Joo Ern Ang, Victoria Revell, Mann Anuska, Simone Mäntele, Daniella T. Otway, Jonathan D. Johnston, Alfred E. Thumser, Debra J. Skene, Florence Raynaud

**Affiliations:** 1 Cancer Research UK Cancer Therapeutics Unit, Division of Cancer Therapeutics, The Institute of Cancer Research, Sutton, Surrey, UK; 2 Faculty of Health and Medical Sciences, University of Surrey, Guildford, Surrey, UK

**Keywords:** Acylcarnitines, Daily variation, Human, Liquid chromatography–mass spectrometry, Metabolomics, Plasma metabolites

## Abstract

Although daily rhythms regulate multiple aspects of human physiology, rhythmic control of the metabolome remains poorly understood. The primary objective of this proof-of-concept study was identification of metabolites in human plasma that exhibit significant 24-h variation. This was assessed via an untargeted metabolomic approach using liquid chromatography–mass spectrometry (LC-MS). Eight lean, healthy, and unmedicated men, mean age 53.6 (SD ± 6.0) yrs, maintained a fixed sleep/wake schedule and dietary regime for 1 wk at home prior to an adaptation night and followed by a 25-h experimental session in the laboratory where the light/dark cycle, sleep/wake, posture, and calorific intake were strictly controlled. Plasma samples from each individual at selected time points were prepared using liquid-phase extraction followed by reverse-phase LC coupled to quadrupole time-of-flight MS analysis in positive ionization mode. Time-of-day variation in the metabolites was screened for using orthogonal partial least square discrimination between selected time points of 10:00 vs. 22:00 h, 16:00 vs. 04:00 h, and 07:00 (d 1) vs. 16:00 h, as well as repeated-measures analysis of variance with time as an independent variable. Subsequently, cosinor analysis was performed on all the sampled time points across the 24-h day to assess for significant daily variation. In this study, analytical variability, assessed using known internal standards, was low with coefficients of variation <10%. A total of 1069 metabolite features were detected and 203 (19%) showed significant time-of-day variation. Of these, 34 metabolites were identified using a combination of accurate mass, tandem MS, and online database searches. These metabolites include corticosteroids, bilirubin, amino acids, acylcarnitines, and phospholipids; of note, the magnitude of the 24-h variation of these identified metabolites was large, with the mean ratio of oscillation range over MESOR (24-h time series mean) of 65% (95% confidence interval [CI]: 49–81%). Importantly, several of these human plasma metabolites, including specific acylcarnitines and phospholipids, were hitherto not known to be 24-h variant. These findings represent an important baseline and will be useful in guiding the design and interpretation of future metabolite-based studies. (Author correspondence: Jooern.Ang@icr.ac.uk or Florence.Raynaud@icr.ac.uk)

## INTRODUCTION

Metabolomics is the study of small molecule (<1 kDa) metabolic profiles in biological systems, and complements genomic and proteomic approaches in providing global views of biological processes. Metabolic profiles capture endogenous and exogenous influences on a living organism and may provide better representation of its functional phenotype than changes in DNA, RNA, and proteins (Allen et al., 2003; Nicholson et al., 2002). Hence, metabolic perturbations caused bysuch disparate factors as genetic changes, microbes, diseases, food, and therapeutic interventions may be investigated using a metabolomic approach (Allen et al., 2003; Nicholson et al., 2002).

Recently published studies have identified clear and pervasive circadian influence on the murine hepatic metabolome (Eckel-Mahan et al., 2012; Fustin et al., 2012). Plasma metabolites in a mouse model that vary significantly with time-of-day have crucially been identified in a landmark study; 14 oscillating metabolites were identified by liquid chromatography–mass spec-trometry (LC-MS) and 28 by capillary electrophoresismass spectrometry (Minami et al., 2009). Direct translation of these animal data to humans, however, cannot be made. In addition to the obvious problem of comparing diurnal and nocturnal species, the timing and amount of feeding, activity, rest, sleep, and posture were not controlled in these animal experiments.

Although daily rhythms regulate multiple aspects of human physiology, rhythmic control of the plasma metabolome remains poorly understood. Previous studies have investigated time-of-day variation in the human metabolome bycomparing metabolomic profilesofmorning with early evening urine samples (Slupsky et al., 2007; Walsh et al., 2006). Although both studies identified significant changes in metabolite levels, such as increased creatinine and dimethylamine in the morning samples, comparison of samples collected at only two time points does not provide comprehensive overview of variation across the 24-h day. Furthermore, these studies did not control for sleep, activity, medication, alcohol/caffeine intake, or environmental lighting, and thus, the results obtained are extremely unlikely to show nonconfounded time-of-day variation. To overcome this, a recent study employed a targeted platform to study the human plasma metabolome under controlled laboratory conditions (Dallmann et al., 2012). The analytical platform, however, was limited to 281 metabolites, and plasma samples from all subjects were pooled at each time point, precluding analyses of intersubject variability and intrasubject daily variation and potentially reducing the sensitivity of this methodology. The current study circumvented these limitations by the use of a global, untargeted metabolomic approach in the analysis of individual plasma samplescollectedfrom healthy human volunteers at different time points. The aim of this proof-of-concept study was characterization of 24-h variation of the plasma metabolome in human subjects maintained under highly controlled conditions of food, posture, light/dark cycle, sleep/wake schedule, and prior exposure to pharmacologic agents. The technological platform with LC-MS usedinthis study has beenpreviously validated by our group with over a thousand metabolites in plasma being reproducibly detected (Pandher et al., 2009, 2012).

## MATERIALS AND METHODS

All aspects of the study were conducted in accordance with the Declaration of Helsinki and conformed to international ethical standards (Portaluppi et al., 2010). A favorable ethical opinion was obtained from the Surrey Research Ethics Committee and the University of Surrey Ethics Committee. Written informed consent was obtained from all participants.

Eligibility for the study was determined via self-completed questionnaires, including General Health Questionnaire, General Sleep Questionnaire, Horne-Östberg Questionnaire, Pittsburgh Sleep Quality Index, Beck Depression Inventory, and Epworth Sleepiness Scale, to assess general health, sleep patterns, and diurnal preference. Full details of the screening process have been previously presented (Otway et al., 2011). To be included, subjects needed to report a regular sleep schedule of between 6 and 8 h in duration and not be extreme morning or evening chronotype according to the Horne-Östberg Questionnaire (Horne & Östberg, 1976). Subjects were excluded if they were taking regular medication or food supplements known to influence metabolism, inflammatory markers, endothelial markers, sleep, or the circadian system, or if they consumed more than four caffeinated beverages per day. Subjects with a history of any of the following were also excluded: (a) circadian or sleep disorder; (b) metabolic, cardiovascular, or chronic infectious/inflammatory disease; (c) psychiatric or neurological disease; and (d) drug and alcohol abuse.

Eight lean, male volunteers were recruited with a mean age of 53.6 (SD ±6.0) yrs, mean body mass index (BMI) of 23.2 kg/m^2^ (SD ±1.4), and fasting glucose of 4.2 mmol/L (SD ±.7). One participant was a smoker, but refrained from smoking for 1 wk prior to study. No participant had undertaken shiftwork within 5 yrs or crossed any time zones within 1 mo of the study. For 1 wk prior to the laboratory study, volunteers were required to maintain scheduled daily meal times (monitored by food diaries) and a fixed sleep/wake schedule (23:00–07:00 h), confirmed via wrist actigraphy (Actiwatch-L; Cambridge Neurotechnology, Cambridge, UK), sleep diaries, and calling a time-stamped voicemail (Otway et al., 2011). Participants also abstained from eating fatty or sugary foods and drinking alcohol or caffeine throughout this baseline week. For the final 3 d of this week, participants were provided with meals of specific nutritional content: the daily calorific content was 1.5-fold the basal metabolic rate (estimated in calories = 11.5 × body weight [kg] + 873), with ∼35% of energy derived from fat (Schofield, 1985).

### In-laboratory Session

The in-laboratory session was conducted at the Surrey Clinical Research Centre. Following an adaptation night in the laboratory, subjects were woken at 06:30 h and commenced a 25-h experimental session throughout which they maintained a semi-recumbent posture to minimize the impact of exogenous factors on the measured parameters. Subjects remained awake in normal room lighting (range 440–825 lux in the direction of gaze) between 06:30 and 22:30 h and were allowed to sleep between 22:30 and 06:30 h in 0 lux. During the waking period, participants were provided with hourly nutritional drinks (Fortisip; Nutricia, Schiphol, The Netherlands) and were allowed to drink water *ad libitum*. The hourly consumption of this drink met the protein, carbohydrate, fat, and fiber requirements of each participant. Daily energy intake was 1.1-fold basal metabolic rate spread equally over the waking hours. Throughout the protocol, including overnight, blood samples were collected via an indwelling cannula by a qualified person, and attempts were made to minimize any disruption of the participants' sleep. Blood samples were collected at selected time points (.5 mL/time point for metabolomic analysis) into lithium heparin tubes, and the plasma fraction was separated by centrifugation (3000 × g, 10 min, 4°C) and stored at –80°C.

### Reagents and Solutions

Water (LC-MS grade), acetonitrile (LC-MS grade), and formic acid (Aristar grade) were purchased from Fisher Scientific (Loughborough, UK), and leucine enkephalin was purchased from Sigma (Poole, UK). External standards, creatine (CAS number: 57-00-1), and colchicine (CAS number: 64-86-8), were purchased from Sigma.

### Plasma Preparation

Samples from selected time points (07:00, 10:00, 16:00, 22:00, 04:00, and 07:00 h [d 2]) were extracted by mixing 1 volume of heparinized plasma with 4 volumes of methanol/ethanol 1:1, followed by centrifugation at 18 000 × g for 15 min at 4°C.

### Assessment of Analytical Variability

Pooled plasma from the eight human subjects from all the sampled time points (i.e., 48 samples) served as quality controls and was analyzed throughout the experimental batch to continuously monitor the analytical variability of the system. These quality-control samples (1/10 injections) were spiked with 1 mM colchicine and creatine. Variability of these spiked compounds and endogenous metabolites, including carnitine, phenyl-alanine, and lysophosphatidylcholine (lysoPC(16:0)), were evaluated.

### LC-MS

Experiments were carried out on two LC-MS systems, namely Acquity UPLC coupled to QTOF Premier mass spectrometer (Waters Corporation, Manchester, UK) and Agilent 1290 Series UPLC connected to a hybrid quadru-pole-time-of-flight Agilent 6510 mass spectrometer (Agilent, Waldbronn, Germany); the first system was used for full-scan analysis and the second system for MS/MS. The robustness, reproducibility, and cross-platform validation of the two systems in studying the exo-metabolome, including plasma, have been previously published (Pandher et al., 2009, 2012). To minimize systematic analytical drift from use of large sample numbers and as the analytical reproducibility of the system is high, one analytical replicate from each individual per time point was used. An electrospray ionization source in positive mode was used for both LC-MS setups in this proof-of-concept study, as the ions detected in the positive mode are known to represent a large proportion of the exo-metabolome in our analytical system (Pandher et al., 2009).

Briefly, chromatographic separation was performed on a Waters Acquity HSS T3 C18 (100 × 2.1 mm, internal diameter [I.D.] 1.8 μm) column. Mobile phase A was LC-MS-grade water containing .1% formic acid and mobile phase B was LC-MS-grade acetonitrile containing .1% formic acid. The column and the autosampler were maintained at a temperature of 50°C and 4°C, respectively. A 13-min linear gradient elution was performed as follows: 100% mobile phase A for the first .5 min, changing to 100% B over 7.5 min, holding at 100% B up to 9.5 min, and finally back to 100% A at 10 min and holding for 3 min. The flow rate was .6 mL/min, with an injection volume of 10 μL. The MS instrument and data acquisition parameters were as previously described (Pandher et al., 2009, 2012).

### Data Handling and Statistical Considerations

Raw data were detected, aligned, and processed using MarkerLynx application manager software (version 4.1; Waters, Milford, MA, USA), with parameters documented previously (Pandher et al., 2009). Each metabolite feature was characterized by a unique combination of mass/charge ratio and retention time. The data matrix obtained was subsequently subjected to multivariate statistical analysis using (i) SIMCA-P v11.0 software (Umetrics AB, Umeå, Sweden): metabolite features that were differentially expressed in one or more of three chosen sets of selected time points—10:00 vs. 22:00 h, 16:00 h vs. 04:00 h, and 07:00 h (d 1) vs. 16:00 h—were identified using orthogonal partial least squares-discriminant analysis (OPLS-DA) with a low threshold of |*p*(corr)| > .5 on the OPLS-DA S-plot (Wiklund et al., 2008); and (ii) repeated-measures analysis of variance (ANOVA) with time as an independent variable; statistical significance was deemed to be achieved at *p* < .05. Extracted ion chromatograms (EICs) of the selected metabolite features were then generated using QuanLynx application manager software (version 4.1; Waters). Finally, cosinor analysis using the mean peak height of EICs of all metabolite features of interest at each time point by the method of least squares (period of 24 h) was carried out to derive estimates by the cosine curve approximation of MESOR (24-h time series mean), amplitude (one-half peak-to-trough variation), acrophase (peak) time, and p value for test of the null hypothesis that the amplitude of the fitted curve was 0; (Nelson et al., 1979); rhythm detection was considered statistically significant when *p* < .05 for the zero-amplitude test.

### Metabolite Identification

The accurate mass and tandem MS fragmentation pattern of each metabolite feature of interest was ascertained and identification performed by database searching (including Human Metabolome Database, Lipid maps, and Metlin) and/or comparison with pure commercial standards. MS/MS was performed on the Agilent system with a default iso-width (width halfmaximum of the quadrupole mass bandpass used during MS/MS precursor isolation) of 4 *m/z* using a fixed collision energy of 15V and data acquired in the range of 50 to 800 Da.

## RESULTS

### Analytical Reproducibility

Within the pooled plasma quality-control samples, coefficients of variation for endogenous metabolites (carni-tine, phenylalanine, and lysoPC(16:0)) were 1.7%, 3.0%, and 8%, respectively, whereas that of spiked exogenous compounds (creatine and colchicine) were 3.6% and 7.7%, respectively.

### Details of Workflow

**FIGURE 1:**
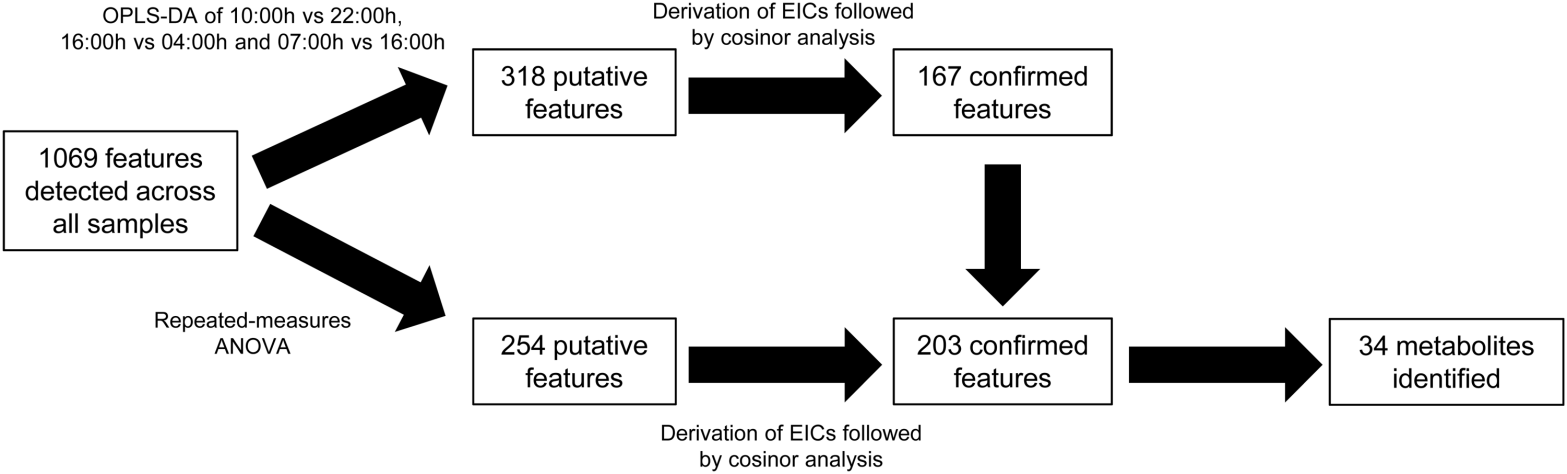
Flowchart summarizing data analysis workflow.

Figure 1 summarizes our data analysis workflow. In the present study, a total of 1069 metabolite features were detected across all analyzed plasma samples. Of these, 318 features passed the OPLS-DA filter using three pair-wise comparisons of 10:00 vs. 22:00 h, 16:00 vs. 04:00 h, and 07:00 vs. 16:00 h. Subsequently, 167 features were confirmed to be significantly 24-h variant using cosinor analysis of EIC data (*p* < .05). In parallel, using repeated-measures ANOVA, 254 putative features were detected, and 203 were confirmed to exhibit 24-h variation. The 203 confirmed features detected by repeated-measures ANOVA represented the sum total of all the temporally variant features detected in this study (19% of all detected features in this study) and included all 167 features identified by OPLS-DA and 36 features additionally identified by repeated-measures ANOVA.

**SUPPLEMENTARY FIGURE 1:**
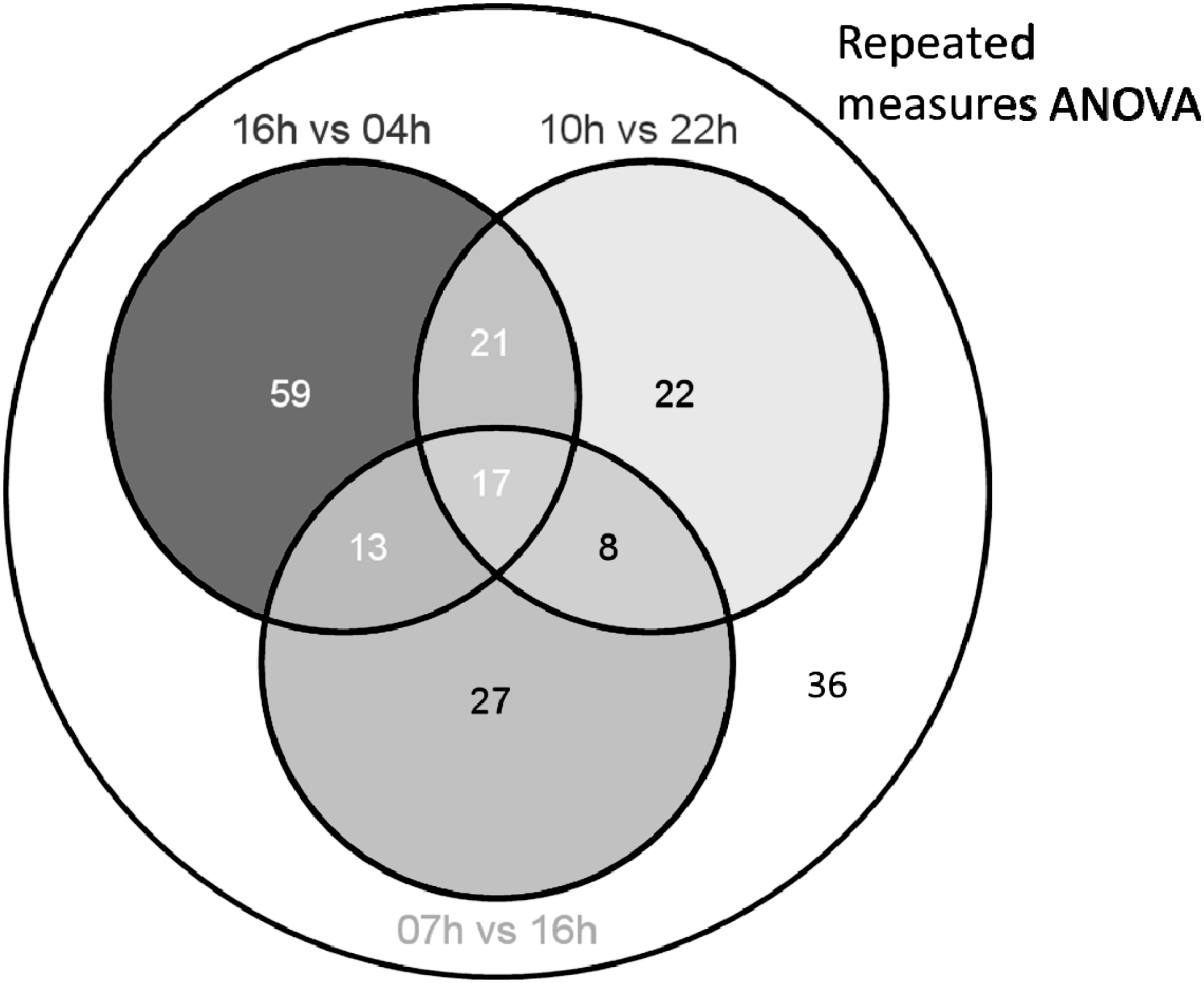
Venn diagram showing overlapping sets of metabolite features detected by OPLS-DA of paired time points and repeated-measures ANOVA across all time points.

Of a total of 203 24-h variant features, the levels of 110 (54%) were significantly different between 16:00 and 04:00 h, whereas those of 68 (33%) and 65 (32%) were significantly different between 10:00 and 22:00 h, and 07:00 and 16:00 h, respectively. Repeated-measures ANOVA detected 36 features unique to those obtained from the stated paired comparisons. These results are summarized in Supplementary Figure 1 and Supplementary Table 1. Using a combination of accurate mass, tandem MS, and online database searches, the identities of 34 metabolites were determined from the 203 rhythmic features. Fragmentation patterns and properties of these compounds on our LC-MS system are summarized in Table 1.

### Variation of Human Plasma Metabolite Levels Across Time-of-Day

Metabolites showing significant 24-h variation were from a variety of chemical classes and included acylcarnitines, lysophospholipids, bilirubin, corticosteroids, and amino acids. For these identified compounds, the mean ratio of oscillation range relative to the MESOR was 65% (95% confidence interval [CI]: 49–81%). Biological variability was consistently greater than analytical variability; the mean analytical coefficient of variation of these ions was 8% (95% CI: 6–10%).

The fitted peak times of the identified 24-h varying plasma metabolites were spread across the day, but appeared to be clustered around early morning, afternoon, and evening (Table 1). Levels of long-chain un-saturated acylcarnitines (C14:1, C14:2, and C18:1) peaked before the long-chain saturated acylcarnitines (C10, C12, C14, C16, and C18) in the morning, whereas the acrophases of short-chain acylcarnitines were observed at radically different times across the day (C2 at 4.9 h, C6 at 5.4 h, C3 at 14.3 h, and C4 at 17.7 h). Bilirubin and cortisol peaked after the start of the light phase, whereas the levels of detected amino acids, such as methionine, tyrosine, proline, lysine, phenylalanine, and leucine, were highest from mid- to late afternoon. By contrast, levels of carnitine, alanine, arginine, tryptophan, and valine (Supplementary Table 1) did not vary significantly over the 24 h. Of the detected phospholipids, lysophosphatidyletha-nolamines (lysoPEs) peaked in the late afternoon and early evening, followed by the phosphatidyl-cholines (PCs), which peaked later in the evening. In contrast, two other phosphocholines (lysoPC(16:0) and lysoPC(18:1)) (Supplementary Table 2) detected in our analytical system had levels that did not change significantly over the day.

**FIGURE 2:**
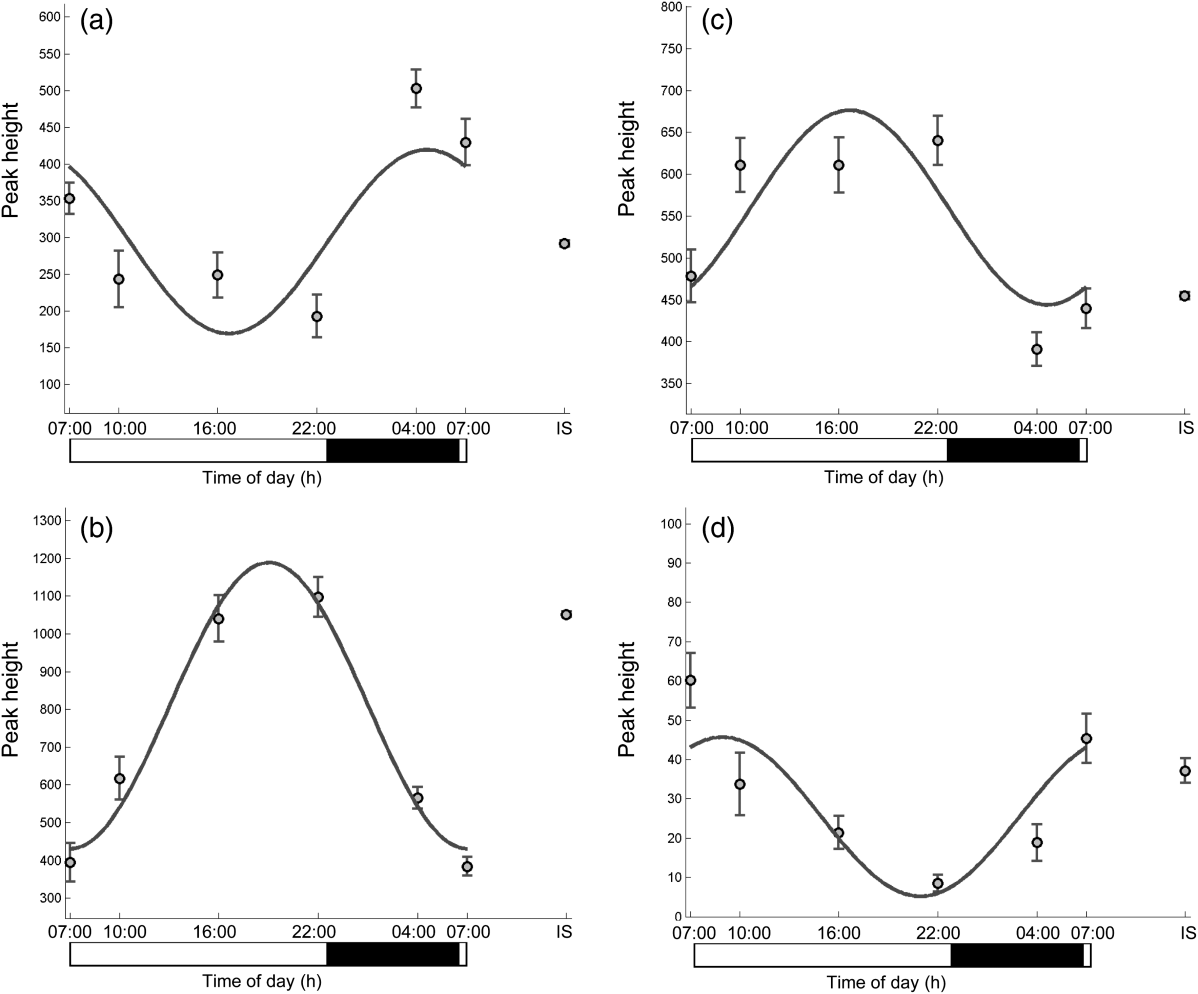
Time profiles of four plasma metabolites with different acrophases: (a) acetylcarnitine, (b) LysoPE(18:1), (c) proline, and (d) cortisol. On the horizontal axis, black bar indicates lights-off (0 lux) and white bar lights-on (440–825 lux). Internal standard (IS) shows the analytical variation of each ion in the pooled, replicate samples analyzed throughout the LC-MS run.

**FIGURE 3:**
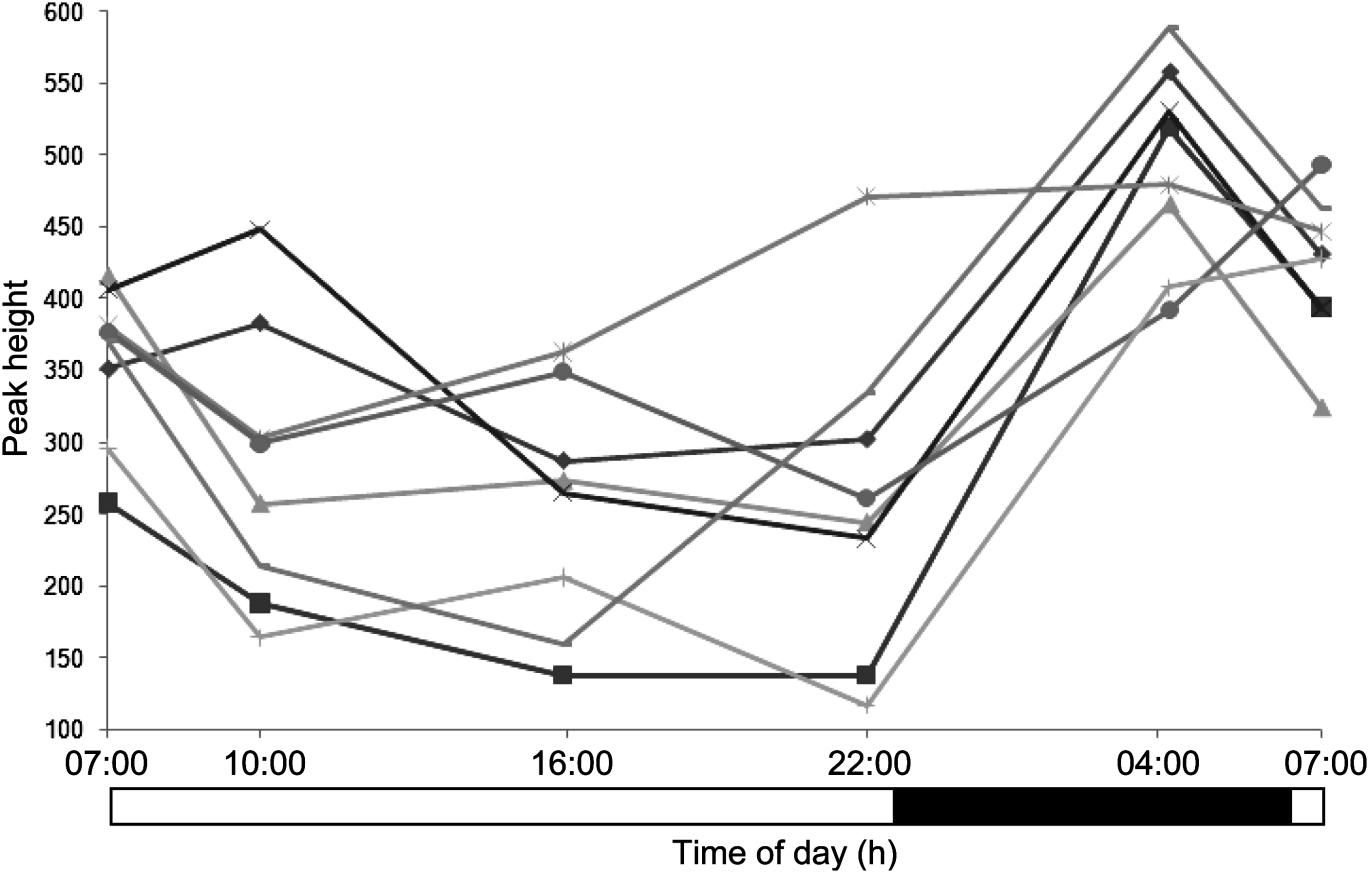
Plasma acetylcarnitine profiles for eight individuals maintained under controlled light/dark, sleep/wake, posture, and calorific intake conditions. Black bar indicates lights-off (0 lux) and white bar lights-on (440–825 lux).

The peak height versus time profiles of four selected metabolites are presented in Figure 2, whereas Figure 3 additionally shows the time profile of one of the metabolites of interest, acetylcarnitine, in each of the eight subjects.

## DISCUSSION

In this study, an untargeted, global metabolomic approach was employed to discover novel 24-h rhythmic metabolites in human plasma obtained under highly controlled conditions. In this context, a UPLC-QTOF MS platform was chosen for its high reproducibility, sensitivity, and mass accuracy. It is stressed that rigorous conditions of sample collection are necessary, as the plasma metabolome is known to be exquisitely sensitive to a broad range of intrinsic and extrinsic factors (Lawton et al., 2008; Lewis et al., 2010; Psychogios et al., 2011). A panel of 24-h rhythmic metabolites from a range of chemical classes were identified, including bilirubin, corticosteroids, amino acids, acylcarnitines, and phos-pholipids. Nineteen percent of the metabolite features detected using our analytical platform exhibited significant daily variation, with the mean oscillation range over MESOR ratio of 65% (95% CI: 49–81%) in the identified metabolites, pointing to the presence of clinically significant time-of-day biological variation.

**TABLE I:** LC-MS characteristics of identified, 24-h variant plasma metabolites and key parameters characterizing temporal variation

Identification	Mass (Da)	Retention time (min)	Analytical CV (%)	Elemental composition	Fragments	Oscillation range/ MESOR (%)	Peak time (dec·h)	*p* value
Octenoylcarnitine (C8:1)	285.20	3.2	4.9	C_15_H_27_NO_4_	286.202, 227.1197, 85.0229, 60.077	7.5	1.4	.020
Acetylcarnitine (C2:0)	203.12	.4	4.2	C_9_H_17_NO_4_	204.124, 85.010, 60.064	94.4	4.9	<.001
Oleoylcarnitine (C18:1)	425.36	6.3	9.7	C_25_H_47_NO_4_	426.358, 85.032, 60.084	43.6	5.2	.039
Hexanoylcarnitine (C6:0)	259.18	2.8	15.1	C_13_H_25_NO_4_	260.181, 201.115, 183.010, 85.029, 60.080	98.8	5.4	<.001
Tetradenenoylcarnitine (C14:1)	369.30	5.2	6.9	C_21_H_39_NO_4_	370.296, 85.029, 60.082	70.8	5.5	<.001
Tetradecadienoylcarnitine (C14:2)	367.28	4.9	11.2	C_21_H_37_NO_4_	368.280, 85.023, 60.076	63.8	6.1	.002
Palmitoylcarnitine (C16:0)	399.34	6.1	17.8	C_23_H_45_NO_4_	400.343, 341.268, 85.029, 60.081	109.8	6.4	<.001
Stearoylcarnitine (C18:0)	427.37	6.7	8.5	C_25_H_49_NO_4_	428.375, 369.304, 85.030, 60.0811	45.6	6.5	.019
Tetradecanoylcarnitine (C14:0)	371.31	5.6	12.8	C_21_H_41_NO_4_	372.309, 313.235, 85.028, 60.081	146.0	6.6	<.001
Dodecanoylcarnitine (C12:0)	343.28	5.0	10.3	C_19_H_37_NO_4_	344.276, 285.201, 133.085, 85.027, 60.079	122.6	7.1	<.001
Bilirubin	584.27	8.2	5.0	C_33_H_36_N_4_O_6_	585.274, 568.243, 299.139, 285.123	51.2	7.1	<.001
Octanoylcarnitine (C8:0)	287.21	3.6	7.4	C_15_H_29_NO_4_	288.213, 85.027, 60.079	68.6	7.4	.003
Decanoylcarnitine (C10:0)	315.25	4.3	9.1	C_17_H_33_NO_4_	316.249, 85.029, 60.080	77.2	7.8	<.001
Cortisol	362.22	3.7	8.1	C_21_H_30_O_5_	363.209, 327.194, 121.063	194.6	8.4	<.001
Cortisone	360.19	3.8	19.5	C_21_H_28_O_5_	361.201, 301.118, 163.071, 105.068	143.0	8.6	<.001
LysoPC(18:4)	515.31	5.5	3.0	C_25_H_46_NO_7_P	516.306, 184.976, 104.107, 86.095	9.8	14.1	.020
Propionylcarnitine (C3:0)	217.14	.5	3.3	C_10_H_19_NO_4_	218.132, 100.986, 85.014, 60.076	28.3	14.3	.009
Methionine	149.06	.5	6.5	C_5_H_11_NO_2_S	150.057, 133.031, 104.054, 61.010, 56.050	20.2	15.3	<.001
Tyrosine	181.08	.5	10.3	C_9_H_11_NO_3_	182.086, 165.051, 136.075, 123.042, 119.050, 91.054	28.4	15.5	<.001
LysoPE(16:0)	453.29	5.9	2.3	C_21_H_44_NO_7_P	454.290, 313.295, 62.058	27.8	16.5	.003
Proline	115.07	.4	3.1	C_5_H_9_NO_2_	116.067, 70.056	47.8	16.6	<.001
LysoPE(18:3)	475.28	5.9	11.2	C_23_H_42_NO_7_P	476.276, 335.269	35.0	16.9	.023
Butyrylcarnitine (C4:0)	231.16	1.9	9.9	C_11_H_21_NO_4_	232.151, 85.026, 60.082	53.4	17.7	<.001
LysoPE(20:4)	501.30	6.1	17.7	C_25_H_44_NO_7_P	502.294, 484.283, 361.274, 62.059	113.2	18.3	<.001
LysoPE(18:2)	477.29	5.7	13.2	C_23_H_44_NO_7_P	478.292, 337.270	110.4	18.8	<.001
LysoPE(18:1)	479.31	6.1	2.2	C_23_H_46_NO_7_P	480.309, 339.289, 62.060	132.0	19.1	<.001
Lysine	146.11	6.8	.1	C_6_H_14_N_2_O_2_	147.113, 130.088, 84.082	7.8	19.1	.014
Phenylalanine	165.09	1.8	2.6	C_9_H_11_NO_2_	166.085, 120.081	18.6	19.3	<.001
LysoPC(18:2)	519.34	5.7	2.5	C_26_H_50_NO_7_P	520.340, 184.073, 104.107, 86.096	44.6	19.6	.001
LysoPC(20:5)	541.32	5.7	2.3	C_28_H_48_NO_7_P	542.324, 524.313, 184.073, 104.107, 86.097	23.7	19.8	.04
LysoPC(20:3)	545.69	6.0	18.6	C_28_H_52_NO_7_P	546.354, 184.072, 104.107, 86.096	57.1	20.0	.02
LysoPC(18:3)	517.32	5.4	5.9	C_26_H_48_NO_7_P	518.324, 184.071, 86.096	67.2	20.5	.002
Leucine	131.10	.5	3.9	C_6_H_13_NO_2_	132.102, 86.098, 44.051	15.6	21.5	.048
LysoPC(20:1) or PC (18:1/2:0)	549.39	6.7	8.9	C_28_H_56_NO_7_P	550.385, 184.070, 104.105, 86.098	26.0	23.1	.020

**SUPPLEMENTARY FIGURE 2:**
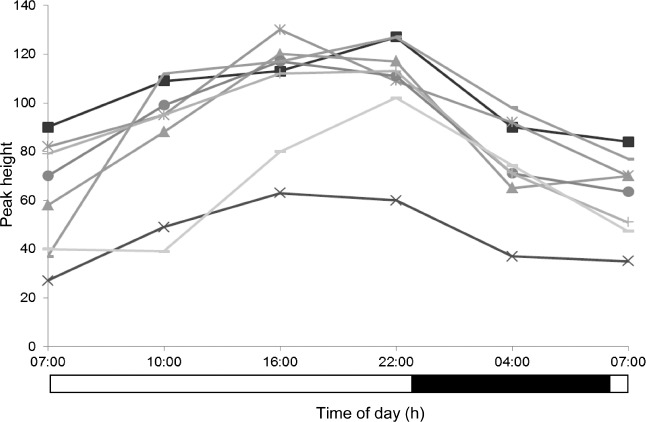
Plasma profiles of metabolite feature with mass/charge 455.19 Da and retention time 5.7 min for eight individuals detected by repeated-measures ANOVA but not pairwise OPLS-DA comparisons, illustrating higher intersubject variability relative to intrasubject time-of-day variation. All participants were maintained under controlled light/dark, sleep/wake, posture, and calorific intake conditions. Black bar indicates lights-off (0 lux) and white bar lights-on (440–825 lux).

In order to identify daily rhythms in metabolites, two sets of time points 12-h apart were selected for comparison, the rationale being they have a periodicity of ∼24 h and typically oscillate in a manner that approximates a cosine curve with the peak and nadir occurring ∼12 h out of phase. To identify such rhythms in metabolites, sets of time points 9–12 h apart were selected for comparison to provide a good probability of detecting something that approximates to the maximum temporal difference. Repeated-measures ANOVA was additionally used to capitalize on the paired nature of the data, i.e., plasma was collected from eight individuals across time. This latter method was the most sensitive and yielded 36 features in addition to OPLS-DA comparisons (Supplementary Figure 1). Not only did this validate results already obtained by OPLS-DA, but it also demonstrated the effect of intersubject variability as well as the relatively higher sensitivity of this approach (Supplementary Figure 1). To illustrate this point, the 24-h temporal profile of a metabolite feature is presented in Supplementary Figure 2, which highlights the significant intersubject variation in this ion compared to intrasub-ject time-of-day changes. In principle, other multivariate tools, such as the multilevel partial least square projection to latent structures (PLS), could be used instead of OPLS-DA for each pairwise comparison in the screening step, as it offers greater statistical power (Westerhuis et al., 2010). In this study, multilevel PLS was not carried out; instead, repeated-measures ANOVA was performed, making use of all data across all time points.

Our data extend previous findings that an unbiased metabolomic platform may be used to identify 24-h variant metabolites (Dallmann et al., 2012; Eckel-Mahan et al., 2012; Fustin et al., 2012; Minami et al., 2009; Slupsky et al., 2007; Walsh et al., 2006); some of the 24-h variant plasma metabolites detected in this study are already known to show such variation, including bilirubin, cortisol, and several amino acids (Eriksson et al., 1989; Feigin et al., 1967, 1968; Larsson et al., 2009; Selmaoui & Touitou, 2003; Wurtman et al., 1968), and this provides further validation of this approach. It is also noteworthy that numerous metabolites linked to major metabolic pathways were identified in this study; the 24-h variant nature of these metabolites has not been previously demonstrated.

Rhythms in human plasma levels of amino acids have been previously reported (Eriksson et al., 1989; Feigin et al., 1967, 1968; Wurtman et al., 1968). Typically, maximal concentrations were observed in the afternoon/evening, and minimal concentrations in the early hours of the morning before waking. Our results are in keeping with these findings: of the detected amino acids, lysine, proline, leucine, methionine, phenylalanine, and tyrosine exhibited significant day/night variation with similar peak/trough temporal changes. The mechanisms regulating this 24-h rhythmicity remain obscure, and they may be related to the periodicity of many other metabolic processes (Eckel-Mahan et al., 2012; Feigin et al., 1971). For instance, the tricarboxylic acid cycle, gluconeogenesis, and lipogenesis utilize amino acid carbon backbones and may be important in this context.

The present study identified numerous plasma acyl-carnitines that demonstrated significant time-of-day variation; only a subset of these has thus far been reported (Dallmann et al., 2012). Acylcarnitines are key intermediates in the β-oxidation of fatty acids in mitochondria (Kompare & Rizzo, 2008), and abnormal levels of these metabolites have been linked to errors of metabolism involving fatty acid oxidation and carnitine cycle (Pasquali et al., 2006). Costa et al. (1999) reported preferential increase in levels of specific mono-unsaturated acylcarnitines in fasting individuals, who generally display increased levels of blood unesterified fatty acids and hepatic β-oxidation. Fasting alone, however, is unlikely to account for the changes observed in the current study, as levels of many of these metabolites peaked and started to decrease in the morning before lights were switched on and feeding of hourly caloric drinks recommenced. Critical components of the biological system regulating the levels of acylcarnitines also demonstrate 24-h variation. For instance, using gene expression profiling data of liver enzymes across the time-of-day in a mouse model, mRNA levels of key transporters of long-chain acylcarnitines, including car-nitine palmitoyltransferase (*CPT*) *1a and 2*, were shown to exhibit clear day/night oscillation (Hughes et al., 2009).

**FIGURE 4:**
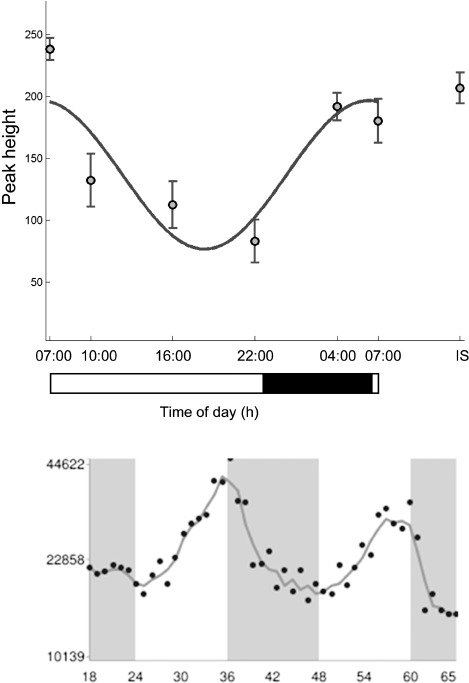
(Top panel) The temporal profile of palmitoylcarnitine in human plasma. (Bottom panel) CPT1a gene expression in a mouse model from an independent study (Hughes et al., 2009). Black/gray bars indicate lights-off and the white bar lights-on.

It is interesting to note that the time-of-day pattern of variation differs between short-chain, unsaturated long-chain, and saturated long-chain acylcarnitines. Levels of long-chain unsaturated acylcarnitines (C18:1, C14:1, and C14:2) peaked early in the morning before the long-chain saturated acylcarnitines (C10, C12, C14, C16, and C18), whereas the acrophases of short-chain acylcarni-tines were observed at radically different times, i.e., C2 at 4.9 h, C6 at 5.4 h, C3 at 14.3 h, and C4 at 17.7 h. Abnormalities in particular enzyme and membrane transporter systems have already been associated with pathognomo-nic changes in acylcarnitine subtypes in well-defined subtypes of inborn errors of metabolism (Santra & Hendriksz 2010). Hence,itisplausible that short- and long-chain acyl dehydrogenases and transporters are regulated differently across time. Indeed, preliminary evidence suggests the presence of such a specific regulatory process. Whereas mRNA levels of *CPT1a* are rhythmic with an acrophase just before lights-off in the mouse—end of the inactivity span, levels of long-chain acylcarnitines transported by this mitochondrial membrane enzyme peak at the end of the dark phase in human subjects—also the end of inactivity span (selected profiles are juxtaposed in time relative to the activity/rest rhythm of the respective species in Figure 4). Given that humans and mice are diurnal and nocturnal species, respectively, agreement of these patterns of temporal change suggest that (*CPT*) *1a* may be responsible for the 24-h variation observed in long-chain acylcarnitines. By contrast, expression profiles of (*CPT*) *1b* and (*CPT*) *1c* did not show significant time-of-day difference in the mouse, lending additional support for the hypothesis that (*CPT*) *1a* may be the specific rhythmic driver of long-chain acylcarnitines (Hughes et al., 2009). It is important to point out that these crossspecies associations, although interesting, are tentative and require further biological validation.

In this study, acrophases of specific C16, C18, and C20 lysoPCs and lysoPEs clustered in the afternoon and evening; no clear exogenous factor related to this could be identified, including food consumption. Two other endogenously expressed plasma phospholipids were assessed and showed no significant time-of-day change, suggesting possible specificity of the rhythmic driver for particular subtypes of lysophospholipids. Of note, temporal variation in plasma lysophospholipid levels has also been detected in mouse plasma (Minami et al., 2009). Enzyme systems currently understood to be mainly responsible for production of these metabolites are the lecithin:cholesterolacyltransferase (Lcat), secreted phospholipase A_2_ (Pla2), and endothelial lipase (Lipg) systems (Yamamoto et al., 2011); in a mouse model, L*cat, Pla2g12a*, and *Lipg* are rhythmically expressed in the liver (Hughes et al., 2009).

The current results will be of use in a number of areas, for example, in the evaluation of pharmacodynamic bio-markers of new drug therapies (Sarker & Workman, 2007). Evaluation of pharmacodynamic biomarkers is made by drawing intrasubject comparisons across time with the patient pre-dose used as a control. This approach, however, may be confounded by the patient's internal circadian and homeostatic systems and external influences, such as dietary intake. The approach taken in the current study typifies a way to validate such pharmacodynamic studies by defining metabolites that are rhythmic and those that are not.

The findings of our study have implications on the use of plasma metabolites in clinical testing. At least 19% of the metabolite features in our study exhibited significant 24-h variation, with the mean oscillation range over MESOR ratio of 65% (95% CI: 49–81%) in the identified metabolites. To improve the predictive utility of clinical biomarkers used in diagnosis, prognosis, and follow-up, determination of 24-h variability should become part of the clinical validation process, especially in the ascertainment of the degree of 24-h variation relative to the factor(s) in question.

A limitation of the present study is that although a global, untargeted approach was adopted, only a proportion of the entire plasma metabolome was monitored using our analytical method. Despite this, the primary objective of identifying and characterizing novel 24-h variant plasma metabolites was met in this proof-of-concept study. Indeed, the present study identified all the 24-h variant metabolites detected by LC-MS in positive ionization mode in the Dallmann et al. (2012) study that was conducted under “constant routine” conditions of constant dim lighting, continuous hourly food supplement, and no sleep, thereby providing external validation of their data. Significantly, additional 24-h variant plasma metabolites were identified in the present study, suggesting that these metabolites may be affected by lighting, sleep, and/or feeding condition. Alternatively, as the samples in the Dallmann et al. (2012) study were pooled for each studied time point, the nondetection of these additional metabolites could be due to lower sensitivity of their approach. Although only eight subjects were studied with six time points across a 24-h day, multiple factors that could confound the rhythms were strictly controlled for. Specifically, study subjects were lean, healthy, and unme-dicated and who maintained a fixed sleep/wake schedule and dietary regime for 1 wk at home prior to an adaptation night, followed by a 25-h session in the laboratory when the light/dark and sleep/wake cycle, posture, and calorific intake were rigorously controlled. In this context, it would be of interest to disentangle the impact of food, sleep, and darkness, and prospective clinical studies are ongoing to address this. In addition, the study participants were all middle-aged men, and the applicability of these findings to females and other age groups is unclear.

In summary, we have successfully applied the use of an untargeted metabolomic approach to identify and characterize 24-h variant metabolites from a range of chemical classes. These findings represent an important baseline and will be useful in guiding the proper design and interpretation of future metabolite-based studies.

## SUPPLEMENTARY

**SUPPLEMENTARY TABLE 1:** List of metabolite features detected in the screening process and considered for tandem MS

Metabolite feature Retention time (min)	Mass/charge (Da)	16h vs. 04h	10h vs. 22h	Comparison made 07h vs. 16h	Repeated-measures ANOVA
0.31	198.94	0	1	0	1
0.31	200.97	1	0	0	1
0.33	288.92	0	1	0	1
0.34	327.05	1	0	0	1
0.34	566.89	0	1	0	1
0.34	634.88	1	0	0	1
0.34	498.9	1	0	0	1
0.38	200.86	0	0	0	1
0.38	134.02	1	0	0	1
0.39	164.03	0	1	0	1
0.41	103.01	1	0	0	1
0.41	116.07	1	0	0	1
0.41	117.08	0	0	0	1
0.41	219.03	0	0	0	1
0.41	221.01	0	0	0	1
0.41	365.11	1	0	1	1
0.41	430.7	1	0	0	1
0.41	204.12	1	0	1	1
0.43	218.14	1	0	0	1
0.43	229.16	1	0	1	1
0.43	335.92	0	0	1	1
0.44	133	1	0	0	1
0.44	194.02	1	0	0	1
0.46	135	1	0	0	1
0.46	150.06	1	0	0	1
0.47	182.08	1	0	0	1
0.49	149.02	1	0	1	1
0.49	132.1	0	1	0	1
0.5	337.02	1	0	0	1
0.55	226.05	1	0	0	1
0.57	254.16	0	0	1	1
0.62	168.99	1	0	0	1
1.66	254.16	0	1	1	1
1.81	103.05	1	0	0	1
1.81	107.05	1	0	0	1
1.81	120.08	0	1	0	1
1.81	131.05	1	1	0	1
1.81	149.06	0	1	0	1
1.81	166.09	1	1	0	1
1.93	232.16	1	0	0	1
2.57	257.17	1	1	1	1
2.58	235.18	1	1	1	1
2.82	260.18	1	0	0	1
2.85	251.18	1	0	1	1
3.12	185.12	1	0	0	1
3.23	286.2	1	0	0	1
3.59	310.2	0	1	0	1
3.62	288.22	0	0	1	1
3.66	363.22	0	1	1	1
3.8	361.2	0	1	1	1
4.17	585.27	0	1	1	1
4.2	243.63	0	0	0	1
4.21	464.28	0	0	0	1
4.24	252.63	0	0	0	1
4.24	412.28	1	0	0	1
4.24	430.3	1	0	0	1
4.24	448.31	0	0	0	1
4.24	488.3	1	0	0	1
4.24	504.27	0	0	0	1
4.24	510.28	0	0	0	1
4.32	316.25	0	0	1	1
4.46	416.32	1	0	0	1
4.46	552.24	0	0	0	1
4.56	470.29	0	0	0	1
4.75	583.26	0	0	1	1
4.79	244.27	0	0	0	1
4.81	207.62	0	0	0	1
4.81	226.63	1	0	0	1
4.81	235.63	0	0	0	1
4.81	244.63	1	0	0	1
4.81	414.3	1	0	0	1
4.81	432.31	1	0	0	1
4.81	450.32	0	0	0	1
4.81	472.3	0	0	0	1
4.81	488.27	1	0	0	1
4.81	494.29	1	0	0	1
4.81	504.24	1	0	0	1
4.82	368.28	0	0	1	1
4.84	344.28	0	0	1	1
4.88	251.12	0	0	0	1
4.92	450.32	1	0	0	1
4.92	472.3	1	0	0	1
4.98	415.21	1	1	0	1
4.99	286.14	1	0	0	1
4.99	437.19	1	0	0	1
4.99	453.17	1	1	0	1
5.16	387.19	0	0	1	1
5.16	409.18	0	0	1	1
5.16	425.15	0	0	0	1
5.18	370.29	0	0	1	1
5.22	432.24	1	1	0	1
5.24	107.09	0	1	0	1
5.24	119.09	1	1	0	1
5.24	135.08	1	1	0	1
5.24	160.04	1	1	0	1
5.24	227.08	1	1	0	1
5.24	233.06	0	1	0	1
5.24	281.14	1	1	0	1
5.24	295.12	1	1	0	1
5.24	300.11	1	1	0	1
5.24	367.14	1	1	0	1
5.24	380.25	0	0	0	1
5.24	397.2	1	0	0	1
5.24	415.21	1	1	0	1
5.24	434.18	1	1	0	1
5.24	437.19	1	1	0	1
5.24	453.17	1	0	0	1
5.24	499.17	1	1	0	1
5.24	129.05	1	0	0	1
5.29	253.63	1	0	0	1
5.29	468.31	0	0	0	1
5.29	490.29	0	0	0	1
5.3	299.14	1	1	1	1
5.3	585.27	1	0	1	1
5.36	476.28	1	1	1	1
5.39	278.64	0	1	0	1
5.39	500.28	1	0	0	1
5.39	518.32	0	1	0	1
5.39	540.31	0	0	0	1
5.42	290.64	1	0	0	1
5.42	564.31	1	0	0	1
5.48	299.14	0	0	1	1
5.48	585.27	0	0	1	1
5.49	494.32	0	1	0	1
5.49	516.31	1	0	0	1
5.49	513.29	1	0	0	1
5.52	643.28	0	1	0	1
5.52	603.3	0	0	1	1
5.55	372.31	1	0	1	1
5.57	337.27	0	0	0	1
5.59	475.79	1	0	0	1
5.61	184.07	0	1	0	1
5.61	385.28	0	0	0	1
5.65	398.33	0	0	0	1
5.67	284.23	0	0	0	1
5.67	516.24	1	1	0	1
5.67	497.24	1	1	1	1
5.68	455.19	0	0	0	1
5.68	460.28	1	1	1	1
5.68	473.2	1	1	1	1
5.68	478.3	1	1	1	1
5.68	500.28	1	1	1	1
5.68	526.3	0	0	0	1
5.68	471.16	0	0	1	1
5.69	548.28	0	0	0	1
5.71	520.34	1	1	1	1
5.71	542.32	0	0	1	1
5.71	590.33	1	0	1	1
5.71	798.97	0	1	0	1
5.71	799.47	0	0	0	1
5.71	279.64	1	0	0	1
5.77	580.3	0	0	1	1
5.85	318.24	0	0	1	1
5.88	313.27	0	0	0	1
5.88	436.28	1	0	0	1
5.88	454.29	1	0	0	1
5.88	476.28	1	0	0	1
5.95	546.36	0	0	0	1
5.96	339.29	1	1	1	1
5.98	400.34	1	1	1	1
6.07	457.2	1	0	1	1
6.07	462.3	1	1	1	1
6.07	475.22	1	1	1	1
6.07	480.32	1	1	1	1
6.07	502.3	1	1	1	1
6.08	520.3	0	0	1	1
6.23	405.26	1	0	0	1
6.27	426.35	1	0	1	1
6.47	428.37	0	0	1	1
6.51	459.32	1	0	0	1
6.54	504.31	0	0	0	1
6.56	273.67	0	0	1	1
6.64	419.28	1	0	0	1
6.71	294.67	0	1	0	1
6.72	550.39	0	1	0	1
6.72	572.37	0	1	0	1
6.82	147.11	0	0	1	1
6.83	650.44	1	1	0	1
6.87	672.42	1	1	0	1
6.93	268.26	1	0	0	1
6.98	279.23	1	1	0	1
7.17	329.25	1	0	1	1
7.27	285.93	0	1	0	1
7.29	350.25	0	1	1	1
7.4	563.55	0	0	0	1
7.54	163.15	0	1	0	1
7.59	455.34	0	0	1	1
7.59	354.2	1	0	1	1
7.59	395.22	0	0	1	1
7.59	561.41	0	0	1	1
7.69	339.18	1	0	1	1
7.7	669.5	1	0	0	1
7.74	284.3	1	0	0	1
7.84	381.3	0	0	1	1
7.92	298.31	0	0	0	1
8.08	780.55	1	0	0	1
8.2	299.14	1	1	1	1
8.2	584.26	0	1	1	1
8.2	585.27	0	1	1	1
8.2	583.26	0	0	1	1
8.21	581.25	0	0	1	1
8.25	389.25	0	1	0	1
8.37	312.33	0	1	1	1

When the feature is significant in a comparison, it is denoted “1” and when it is not, it is denoted “0”.

**SUPPLEMENTARY TABLE 2:** LC-MS features of metabolites of interest that did not exhibit significant 24-h variation (p > .05)

Identification	Retention time (min)	Mass (Da)	Elemental composition	Fragments
Arginine	0.39	174.112	C_6_H_14_N_4_O_2_	175.120, 158.070, 116.050, 70.100
Valine	0.41	117.079	C_5_H_11_NO_2_	118.087, 72.070, 55.040
Carnitine	0.42	161.105	C_7_H_15_NO_3_	162.113, 103.040, 85.030, 60.080
Alanine	0.42	89.048	C_3_H_7_NO_2_	90.056, 44.050
Tryptophan	2.03	204.09	C_11_H_12_N_2_O_2_	205.098, 188.069, 146.059, 118.064
LysoPC(16:0)	5.9	495.333	C_24_H_50_NO_7_P	496.340, 184.074, 104.105, 86.097
LysoPC(18:1)	6.09	521.348	C_26_H_52_NO_7_P	522.356, 184.073, 104.107, 86.095
